# From expansion to intelligent governance: structural transformation and regulatory paradigm reconstruction in China’s health insurance reform: a systematic literature review on the evolution and future trajectories of health insurance fund supervision

**DOI:** 10.3389/fpubh.2026.1904242

**Published:** 2026-10-30

**Authors:** Zeyu Zhu

**Affiliations:** Department of Medical Insurance and Pricing, The First Affiliated Hospital of Guangdong Medical University, Dongguan, China

**Keywords:** China, DRG/DIP, fraud detection, fund supervision, health policy, healthcare financing, intelligent governance, payment reform

## Abstract

**Background:**

Over the past quarter century, China has undergone three transformations in healthcare financing: from employer-sponsored coverage to universal enrollment, then toward quality- and intelligence-oriented governance. This review examines the evolution and future of medical insurance fund supervision in China.

**Methods:**

Five databases were searched on June 15, 2025. Included publications addressed fund supervision, payment reform, intelligent technology, sustainability, equity, or anti-corruption in China. MMAT and AMSTAR-2 criteria were used for quality appraisal. Each publication was independently assessed by two reviewers using the MMAT screening questions and domain ratings; disagreements were resolved through consensus discussion, with an independent third assessor arbitrating unresolved cases. Inter-rater reliability on a 20% random subset yielded substantial agreement (Cohen’s κ = 0.82). PRISMA 2020 guidelines were followed; no PROSPERO registration was made.

**Results:**

Three core findings emerged via the Intelligent Supervision Integration (ISI) framework (data, algorithm, institution layers). First, fund supervision evolved through three phases, with the 2018 NHSA establishment as pivotal. Second, DRG/DIP reforms contained costs but generated upcoding and cost-shifting. Third, ML-based and blockchain-based fraud detection have transitioned to operational deployment.

**Conclusion:**

The ISI framework explains China’s transition to intelligent governance. Six prospective trends are identified: (1) data-driven supervision, (2) federated learning for privacy-preserving analytics, (3) blockchain-based full-chain supervision, (4) intelligent actuarial early warning systems, (5) institutionalized integrity governance, and (6) localization-optimized payment reform.

## 1 Introduction

The evolution of China’s social health insurance system constitutes one of the most rapidly achieved near-universal coverage expansions in recent decades (1). From the inauguration of the Urban Employee Basic Medical Insurance (UEBMI) scheme in 1998, China has constructed a universal insurance network covering more than 1.35 billion individuals within two decades, maintaining an enrollment rate consistently above 95%. In February 2020, the Central Committee of the Communist Party and the State Council issued the Opinions on Deepening the Reform of the Medical Security System, articulating an ambitious target: by 2030, the country aims to establish a multi-tiered healthcare financing architecture—anchored by basic medical insurance, underpinned by medical assistance, and supplemented by commercial health insurance, charitable giving, and mutual aid schemes (2). This policy pronouncement signaled a fundamental recalibration of China’s healthcare reform agenda from a focus on coverage breadth to one centered on quality and effectiveness.

Yet the rapid expansion of enrollment has exposed the insurance fund to mounting security challenges. According to the NHSA (3), nationwide fund supervision inspections covered 289,900 designated medical institutions in 2024 alone, with 2,008 confirmed fraudulent institutions and 3,018 criminal cases jointly investigated with public security authorities; cumulative fund recoveries exceeded CNY 100 billion between 2018 and 2024, with 275 billion yuan recovered in 2024 alone. The question of fund oversight has thus transcended routine financial auditing to become a matter of systemic governance bearing directly on the long-term viability of the entire insurance apparatus.

This review makes three scholarly contributions. First, it delineates the three-phase historical trajectory of Chinese health insurance fund supervision from 1998 to the present, revealing the internal logic of institutional change. Second, it integrates three seemingly disparate research domains—payment reform, fund oversight, and intelligent governance—into a unified analytical framework, advancing the central thesis that China is undergoing a data-driven paradigm shift in fund regulation. Third, drawing on the most recent academic literature, it offers a prospective analysis of six emerging trends that will likely shape the future trajectory of insurance fund supervision in China.

This review systematically synthesizes evidence on the structural transformation of China’s health insurance fund supervision, examining how payment reform, intelligent technology, sustainability, equity, and anti-corruption intersect within a unified governance paradigm. These six thematic domains are not arbitrarily combined but represent the interdependent dimensions of a single regulatory challenge: payment reform reshapes provider incentives (fund-side), intelligent technologies empower oversight (technology-side), sustainability and equity constitute regulatory objectives (outcome-side), and anti-corruption provides the institutional means (governance-side). The Intelligent Supervision Integration (ISI) framework serves as the integrative logic connecting these dimensions.

## 2 Literature review

Although the preceding sections reference key studies in passing, a dedicated review of existing systematic and narrative syntheses is necessary to situate the present review’s contribution. Prior reviews of China’s health insurance reforms have largely focused on single dimensions—coverage expansion, payment mechanism design, or specific anti-fraud technologies—without integrating these strands into a unified analytical framework. Table 1 summarizes the most pertinent existing reviews and identifies the gaps this review seeks to address.

**Table 1:** Comparison of existing reviews and identification of research gaps.

Author (year)	Journal	Focus/Scope	Key findings	Gap/Limitation
Yip et al. (2019) (1)	Lancet	10-year progress in UHC	Achieved >95% coverage; gaps in service quality and financial protection	Single dimension (coverage); no fund supervision or technology analysis
Meng et al. (2015) (4)	Lancet	Consolidation of insurance schemes	Fragmentation exacerbated inequities; integration needed	Pre-NHSA; no intelligent governance or payment reform analysis
du Preez et al. (2025) (36)	Artif Intell Med	ML for healthcare fraud detection (global)	RF and XGBoost most used; GNNs and FL are frontier	Global scope; no China-specific institutional context
Al-Jarrah et al. (2025) (34)	Information	Deep learning in insurance fraud	DL outperforms conventional ML on imbalanced data	Technical survey only; no policy or governance dimension
Behzadi et al. (2026) (60)	Health Econ Rev	Provider behavioral responses to PPS (WHO framework)	Behavioral responses universal; context matters	International; no China-specific DRG/DIP or fund oversight
Wang et al. (2025) (18)	Front Public Health	DIP payment reform in Guangzhou	DIP reduced costs; operational simplicity	Single-city, single payment mechanism; no supervision technology
Shi et al. (2018) (57)	BMJ Open	Pharmaceutical corruption reforms	Mixed-method analysis of bribery remedies	Anti-corruption only; no payment reform or technology integration

As Table 1 demonstrates, existing reviews consistently focus on isolated dimensions of China’s health insurance system. No prior review has integrated payment reform, intelligent supervision technology, fund sustainability, equity, and anti-corruption governance within a single analytical framework. This review addresses that gap by (i) spanning all six thematic domains, (ii) employing the ISI framework to map cross-domain relationships, and (iii) incorporating both English- and Chinese-language evidence to capture the full policy landscape. The broad scope is therefore not a weakness but a deliberate design choice grounded in the recognition that these dimensions are structurally interdependent within China’s evolving governance paradigm.

## 3 Methods

### 3.1 PICO(P)S statement

The research question was formulated using the PICO(P)S framework as follows. Population (P): the Chinese social health insurance system, including its supervised entities (designated medical institutions, insured populations, and administrative agencies) from 1998 to 2025. Intervention (I): health insurance fund supervision mechanisms, encompassing payment reforms (DRG/DIP, global budgeting), intelligent monitoring technologies (machine learning, blockchain, federated learning), and integrity governance frameworks. Comparator (C): historical baselines (pre-reform periods, pre-2018 NHSA era), cross-regional variations in reform implementation, and alternative regulatory approaches. Outcomes (O): fund security and sustainability, provider behavioral responses (upcoding, cost-shifting, patient selection), healthcare quality and access equity, catastrophic health expenditure reduction, and anti-corruption effectiveness. Study design (S): peer-reviewed empirical studies employing randomized controlled trials, quasi-experimental designs (difference-in-differences, interrupted time series, regression discontinuity), longitudinal and cross-sectional analyses, systematic reviews and meta-analyses, qualitative and mixed-methods studies, and policy documents issued by the National Healthcare Security Administration and the State Council.

The broad scope encompassing six thematic domains is justified by their structural interdependence within China’s fund supervision system: payment reform (DRG/DIP) directly shapes provider incentives that supervision must address; intelligent technologies provide the monitoring tools; sustainability and equity constitute the dual objectives of supervision; and anti-corruption governance provides the institutional backbone. Narrowing the scope to any single dimension would fail to capture the systemic nature of the regulatory transformation this review seeks to analyze.

### 3.2 Study design

This review employed a systematic review design with narrative synthesis, following the Preferred Reporting Items for Systematic Reviews and Meta-Analyses (PRISMA) 2020 guidelines. Both quantitative and qualitative study designs were eligible for inclusion, reflecting the multi-faceted nature of health insurance fund supervision research, which spans health economics, public health policy, data science, and institutional analysis. A meta-analysis was not conducted due to the substantial heterogeneity in study designs, outcome measures, and intervention contexts across the included literature.

### 3.3 Search strategy and literature identification

A systematic literature search was conducted across five electronic databases: PubMed/MEDLINE, EMBASE, Web of Science Core Collection, the China National Knowledge Infrastructure (CNKI), and the Wanfang Data Knowledge Service Platform. Searches were performed on June 15, 2025 using the following core MeSH terms and keywords: “health insurance fund”; “medical insurance supervision”; “payment reform”; “DRG”; “DIP”; “diagnosis-related groups”; “intelligent supervision”; “fraud detection”; “healthcare fraud”; “fund sustainability”; “anti-corruption”; “China” Chinese-language searches on CNKI and Wanfang employed the following policy-specific terms: 飞行检查 (unannounced inspection), 欺诈骗保(insurance fraud and deception), 医保基金监管 (medical insurance fund supervision), 按病种分值付费 (DIP), 智能审核、智能监控 (intelligent auditing, intelligent monitoring), and 结余留用 (surplus retention). Supplementary Boolean operators (AND/OR) were applied to combine domain-specific terms. No date restriction was imposed on the lower bound; the upper limit was set at June 2025. Both English-language and Chinese-language publications were eligible for inclusion.

Full search strategies for all five databases, including complete Boolean query strings, field restrictions, filters, search dates, and initial hit counts, are provided in Supplementary Table S1 to ensure full reproducibility of the search process.

### 3.4 Inclusion and exclusion criteria

Publications were included if they: (1) addressed health insurance fund supervision, payment reform, intelligent regulatory technology, fund sustainability, coverage equity, or anti-corruption mechanisms in the context of China’s healthcare system; (2) were peer-reviewed empirical studies, systematic reviews, policy documents, or seminal theoretical works; (3) were published in English or Chinese. Publications were excluded if they: (1) focused exclusively on clinical treatment protocols without relevance to fund governance or payment systems; (2) were conference abstracts, editorials, letters, or unpublished manuscripts; (3) were duplicates of included studies; or (4) lacked sufficient methodological detail for quality assessment.

### 3.5 Study selection and data extraction

Database searches yielded an initial pool of 412 records. After deduplication (*n* = 63 removed), 349 titles and abstracts were screened. Of these, 197 were excluded as irrelevant. Full texts of 152 potentially eligible articles were assessed, of which 91 were excluded for the following reasons: not peer-reviewed (*n* = 24); insufficient focus on China’s health insurance system (*n* = 38); or duplicated findings from other included studies (*n* = 29). A total of 61 sources were ultimately included in this narrative synthesis. Data extraction covered: study design, setting, sample size or data source, main outcomes, and relevance to one or more of the six thematic domains of this review. Quality appraisal for primary studies followed the Mixed Methods Appraisal Tool (MMAT) criteria; systematic reviews were assessed using AMSTAR-2 criteria.

Title and abstract screening was performed by the primary reviewer, with a second reviewer independently verifying uncertain cases. Full-text assessment and data extraction were conducted independently by two reviewers. Disagreements were resolved through consensus discussion; where consensus was not reached. Inter-rater reliability for full-text inclusion was assessed on a random 20% subset (n = 30 articles), yielding substantial agreement (Cohen’s *κ* = 0.82). A standardized data extraction form was piloted on five articles and refined before full application.

### 3.6 Risk of bias assessment

Risk of bias and methodological quality were assessed using two validated instruments appropriate to study design. For primary empirical studies, the Mixed Methods Appraisal Tool (MMAT) version 2018 was applied, covering five design categories (qualitative, quantitative randomized controlled, quantitative non-randomized, quantitative descriptive, and mixed methods) with two screening questions and five quality criteria per category. For systematic reviews, AMSTAR-2 (A Measurement Tool to Assess Systematic Reviews) was employed, evaluating 16 domains including protocol registration, comprehensive literature search, duplicate study selection and data extraction, risk of bias assessment, and publication bias analysis. Each included publication was independently assessed by two reviewers using the MMAT screening questions and domain ratings; all disagreements were resolved through consensus discussion, with. Studies were categorized as high, moderate, or low quality based on the number of criteria fully satisfied. No study was excluded solely on the basis of quality rating; rather, low-quality studies were retained where they provided unique policy insights, with sensitivity analyses conducted to assess their influence on synthesis conclusions (see Section 4.2 for results).

Study-level MMAT and AMSTAR-2 quality assessment results, including individual criterion ratings for each included publication, are provided in Supplementary Table S2.

### 3.7 Data synthesis and analysis

Due to the substantial heterogeneity in study designs (ranging from randomized controlled trials and quasi-experimental evaluations to qualitative case studies and policy analyses), outcome measures (cost containment, equity indices, provider behavior, fraud detection accuracy, fund sustainability projections), and intervention contexts (geographic regions, institutional settings, reform stages), a meta-analysis was not feasible. A narrative synthesis approach was adopted, following the Economic and Social Research Council (ESRC) guidance on narrative synthesis. Included publications were grouped into six thematic domains (historical evolution, payment reform co-adaptation, intelligent supervision technologies, fund sustainability, equity and protective effects, and anti-corruption governance), and findings were synthesized both within and across domains. Cross-domain integration was facilitated by the Intelligent Supervision Integration (ISI) analytical framework, which maps findings across data, algorithm, and institutional layers.

The six thematic domains were derived through a combined deductive–inductive process: the initial deductive framework was based on the PICO(P)S components, while the final domain structure was refined inductively through iterative reading of included publications. Within each domain, evidence was clustered by study design, outcome type, and geographic/institutional context to facilitate pattern identification. Where findings from different studies were discrepant (e.g., divergent directions of DRG impact on costs across hospital tiers), a structured heterogeneity analysis was conducted—grouping studies by hospital level, region, reform stage, and measurement approach—to identify potential explanatory factors. These discrepancies and their possible explanations are explicitly noted in the relevant Results subsections. The narrative synthesis is reported in accordance with the Synthesis Without Meta-analysis (SWiM) reporting guideline.

### 3.8 Reporting guidelines and protocol registration

This systematic review was conducted and is reported in accordance with the Preferred Reporting Items for Systematic Reviews and Meta-Analyses (PRISMA) 2020 guidelines (see Supplementary File). The PRISMA 2020 checklist was completed and is available as a Supplementary document. No review protocol was prospectively registered on PROSPERO or other platforms prior to commencement, as acknowledged in Section 5.3 (Limitations).

## 4 Results

### 4.1 Study selection and PRISMA flow

The PRISMA 2020 flow diagram detailing the study selection process is presented in Figure 1. Of the 412 records initially identified through database searches, 63 duplicates were removed, leaving 349 unique records for title and abstract screening. Following screening, 197 records were excluded as not relevant to health insurance fund supervision in China. Full-text assessment was conducted for 152 potentially eligible publications, of which 91 were excluded (not peer-reviewed: *n* = 24; insufficient focus on China’s health insurance system: *n* = 38; duplicated findings: *n* = 29). A total of 61 publications were ultimately included in the narrative synthesis.

**Figure 1:**
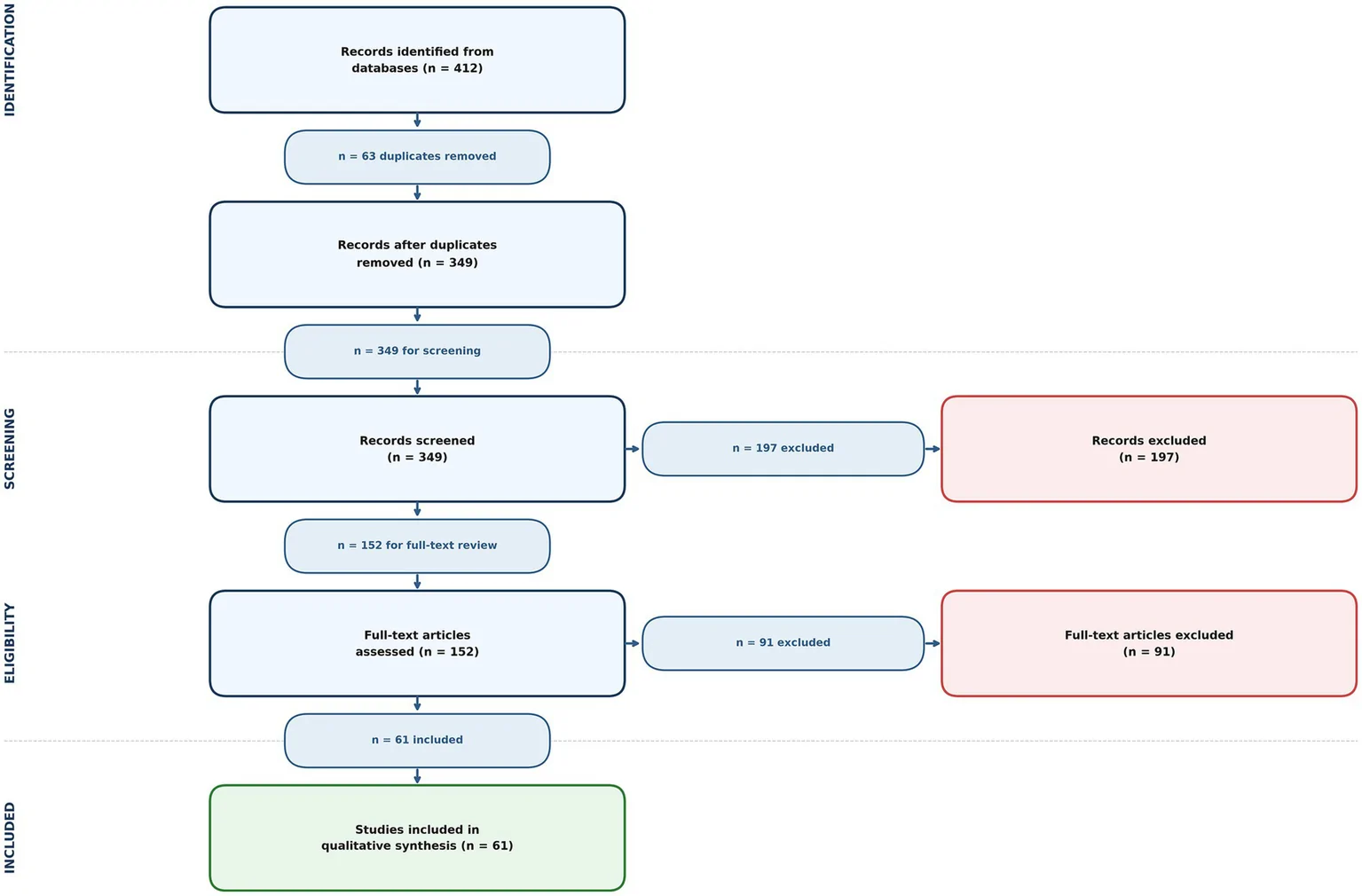
PRISMA 2020 flow diagram of study selection. Of 412 records identified, 63 duplicates were removed, 349 records were screened, 197 were excluded after title/abstract review, 152 full-text articles were assessed for eligibility, and 91 were excluded with reasons, yielding 61 publications for inclusion in the final synthesis.

### 4.2 Risk of bias assessment results

Among the 61 included publications, methodological quality varied across study designs and domains (Supplementary Table S2). Forty-three publications (70.5%) were rated as high quality, meeting all or nearly all MMAT or AMSTAR-2 criteria applicable to their design. Thirteen publications (21.3%) were rated as moderate quality, typically due to incomplete reporting of confounding control in quantitative studies or insufficient reflexivity in qualitative studies. Five publications (8.2%) were rated as low quality, primarily due to unvalidated outcome measures or inadequate sampling strategies; these were retained because they provided unique policy insights (e.g., institutional analyses of DRG/DIP implementation in single-province settings). A sensitivity analysis excluding the five low-quality publications did not materially alter the synthesis conclusions, confirming the robustness of the review findings.

Detailed study-level quality assessment results, including individual MMAT criterion ratings and AMSTAR-2 domain evaluations for each included publication, are provided in Supplementary Table S2.

### 4.3 Historical evolution of China’s health insurance fund supervision: a three-phase institutional analysis

#### 4.3.1 Phase I: Institutional establishment and decentralized management (1998–2009)

The modern Chinese healthcare financing system traces its origins to the 1998 State Council Decision on Establishing the Urban Employee Basic Medical Insurance System (Guo Fa [1998] No. 44), which codified a hybrid model combining social pooling accounts with individual savings accounts. This decree represented a decisive break from the planned-economy era of employer-provided healthcare toward a modern social insurance paradigm.

During this initial phase, fund oversight exhibited a characteristically fragmented profile. UEBMI and subsequently URBMI, launched in 2007, fell under the jurisdiction of the Ministry of Human Resources and Social Security, whereas the New Cooperative Medical Scheme (NCMS), inaugurated in 2003, was administered by the Ministry of Health. Medical assistance programs were further managed by the Ministry of Civil Affairs. This urban–rural administrative divide produced systemic dysfunctions: inconsistent enforcement standards, pervasive information silos, and chronic difficulties in cross-agency coordination (3).

In a landmark study published in The Lancet, Meng et al. (4) conducted a systematic evaluation of China’s social health insurance consolidation process, documenting how the three coexisting insurance schemes diverged sharply in contribution levels, reimbursement ratios, and benefit scopes. This “fragmentation” dynamic not only exacerbated health inequities but also imposed prohibitive costs on fund supervision.

Wang et al. (5) provided longitudinal evidence from a per-capita payment reform pilot in Shanghai’s Pudong New Area, demonstrating that grassroots payment innovations—while achieving some cost containment—were fundamentally constrained by the absence of cross-system data infrastructure and a unified regulatory architecture.

#### 4.3.2 Phase II: Universal coverage and system integration (2009–2018)

Following the launch of the 2009 healthcare reform, China achieved over 95% population coverage within a single decade (1). The policy focus accordingly shifted from broadening enrollment to integrating the disparate insurance programs.

Li et al. (6), in a comprehensive assessment of China’s primary healthcare system also published in The Lancet, noted that the 2009 reform pursued a three-pronged strategy—expanding insurance coverage, establishing an essential medicines system, and strengthening primary care delivery. While this approach succeeded in achieving near-universal insurance enrollment, the authors highlighted that formal coverage did not automatically translate into equitable access: rural residents, internal migrants, and older adult populations continued to exhibit significantly lower utilization rates compared with urban employees.

Diao and Liu (7) examined the equity of benefit distribution across China’s basic medical insurance schemes, revealing a stark discrepancy between formal enrollment rates exceeding 95% and substantial gaps in actual benefit levels across insurance categories. Urban employees consistently received higher reimbursement proportions and broader coverage than their rural and informal-sector counterparts, exposing a profound gulf between de jure equality and de facto equity.

Wang et al. (8) further corroborated these findings, demonstrating that rural–urban insurance integration reduced the incidence of catastrophic health expenditures only modestly, and that the effectiveness of integration was moderated by persistent differentials in funding levels and reimbursement policies across regions.

On the institutional front, this period witnessed the introduction of insurance fund budgetary systems, strengthened service agreement management, and exploratory payment reform pilots. Nevertheless, the persistent “multi-agency” governance structure continued to impose structural constraints on regulatory effectiveness.

#### 4.3.3 Phase III: NHSA and comprehensive governance (2018–present)

In March 2018, the First Session of the 13th National People’s Congress approved the establishment of the National Healthcare Security Administration (NHSA), consolidating insurance management functions previously dispersed across the Ministry of Human Resource and Social Security, the National Health Commission, the National Development and Reform Commission, and the Ministry of Civil Affairs. This bureaucratic reorganization has been characterized by scholars as a “historical turning point” in the modernization of China’s healthcare governance (9).

Gong et al. (9), writing in Frontiers in Public Health, analyzed the strategic equilibrium among pharmaceutical, healthcare provider, and insurance actors in China’s coordinated reform, arguing that the NHSA’s creation fundamentally altered the balance of power. The insurance side transitioned from a fragmented and passive payer to a centralized strategic purchaser—a transformation with decisive implications for the strengthening of fund oversight.

Yang and Yang (10), also in Frontiers in Public Health, drew on qualitative fieldwork from China’s healthcare reform pilots to identify the conditions under which policy experiments succeed or fail. They found that the NHSA’s post-2018 initiatives—including centralized volume-based procurement, DRG/DIP payment reform, and smart supervision platform development—achieved traction largely because they overcame the information barriers and institutional entanglements that had previously characterized sectoral governance.

Yue et al. (11) employed spatiotemporal modeling to assess the coupling coordination among China’s “three medical systems” (三医联动, the coordinated reform of medical insurance, healthcare delivery, and pharmaceuticals), finding that coordination levels improved markedly after the NHSA’s establishment, laying the organizational foundation for comprehensive fund governance.

The enactment of the Medical Insurance Fund Supervision Regulation (State Council Decree No. 735) in 2021 represented a further milestone, elevating fund oversight from administrative guidelines to statutory law. This regulation established a tripartite framework encompassing agreement management, administrative enforcement, and judicial accountability.

Shen et al. (12), drawing on judicial data, analyzed the institutional impact of China’s healthcare anti-corruption campaign, demonstrating that the NHSA’s creation generated synergies with enforcement actions, amplifying the institutional pressure on healthcare organizations to operate in compliance with regulatory norms.

The three-phase evolution of China’s medical insurance fund supervision system is illustrated in Figure 2, which depicts the key milestones, institutional characteristics, and supervision gaps for each phase.

**Figure 2:**
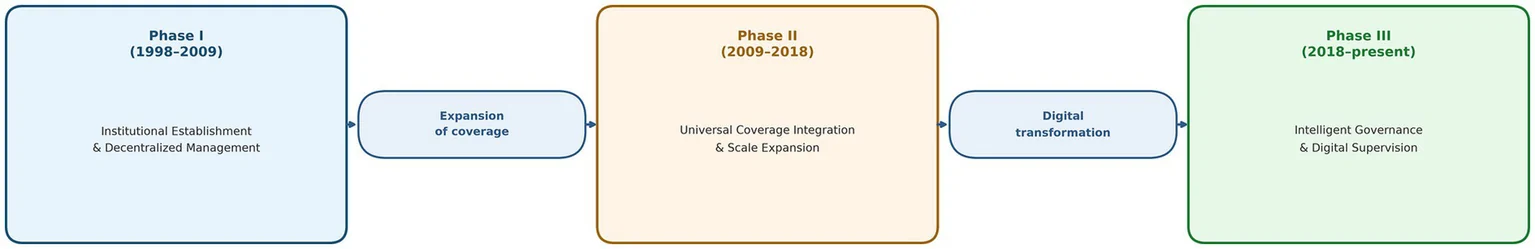
Three-stage evolution of China’s medical insurance fund supervision system: from decentralized management (1998–2009) through universal coverage integration (2009–2018) to comprehensive governance (2018–present). Key milestones, institutional characteristics, and supervision gaps are annotated for each phase. Figure has been redesigned so that transitional text between phases is embedded within the arrow shapes rather than floating outside, improving readability.

### 4.4 Co-adaptation of payment reform and fund oversight

#### 4.4.1 From fee-for-service to global budgeting

The initial move away from fee-for-service (FFS) payment in China took the form of global budgeting, which was designed to displace FFS—widely regarded as the root-cause driver of escalating healthcare expenditures (13).

Zhang et al. (14), using experimental methods, assessed the impact of global budgets on healthcare delivery efficiency. Their findings confirmed that global budgeting significantly reduced per-admission costs and average length of stay, but also induced undesirable behaviors—including the refusal to admit severely ill patients and the artificial splitting of hospitalizations—suggesting that cost-control instruments require complementary quality assurance mechanisms.

He et al. (15), employing a difference-in-differences approach, evaluated the effects of global budgeting on expenditure containment and readmission patterns. While cost control was effective in the short run, the impact attenuated over time as hospitals learned to adapt through coding adjustments and service fragmentation.

Zhou et al. (16) examined whether combining global budgets with pay-for-performance (P4P) mechanisms could mitigate potential quality deterioration in county-level hospitals. Their evidence indicated that P4P partially compensated for quality erosion, though the design of performance indicators required further refinement.

#### 4.4.2 Institutional innovation and challenges of DRG/DIP reform

The DRG and DIP represent the two principal instruments of China’s payment reform agenda. DRG, originating in the United States, groups patients by clinical similarity and resource consumption intensity. DIP, an indigenously developed Chinese approach, operates through a point-scoring system tied to regional global budgets, offering greater adaptability to China’s uneven distribution of medical resources.

The technical differences between DRG and DIP extend beyond their historical origins and carry profound implications for provider behavior and fund oversight. Three distinctions merit emphasis. First, the grouping logic is fundamentally different. DRG adopts a “group first, price later” approach, compressing tens of thousands of diagnosis–procedure combinations into 600–1,000 clinically coherent groups, each assigned a predetermined weight and payment rate. DIP, by contrast, follows a “price first, classify later” logic, cross-tabulating ICD-10 diagnoses with ICD-9-CM-3 procedures to generate tens of thousands of case-type combinations, each carrying a point value that is converted into monetary terms only at year-end settlement. Second, payment certainty diverges sharply. Under DRG, the payment rate for each group is fixed at the beginning of the fiscal year, giving hospitals a predictable revenue envelope. Under DIP, however, the monetary value of each point depends on the regional global budget divided by the total number of points generated across all hospitals in the region—meaning individual hospitals cannot know their actual unit revenue until after the settlement period closes. This structural uncertainty is a core institutional feature of the DIP system and the root cause of many hospitals reporting that they “only know how much they earned at year-end.” A side-by-side comparison of DRG and DIP is provided in Table 2.

**Table 2:** Comparative analysis of DRG and DIP payment reform models in China.

Dimension	DRG (diagnosis-related groups)	DIP (diagnosis-intervention packet)
Origin	United States (Yale, 1970s)	China (original design,2018)
Grouping logic	Clinical similarity + resource consumption similarity	Disease category scores + regional global budget
Payment unit	Fixed rate per DRG group	Point value per disease intervention
Cost control effect	Significant; compresses spending distribution (20)	Moderate; 1.25–3.42% reduction in total costs (21)
Length of stay effect	Significantly reduced (Liu et al.,2026)	Significantly reduced (22)
Patient OOP trend	Increased (+0.82%/month, *p* < 0.01) (19)	Mixed evidence; depends on local implementation
Unintended behaviors	Upcoding, low-value hospitalization, cherry-picking (25)	Cost shifting to consumables (55.83%contribution) (21)
Implementation complete	High; requires sophisticated coding and IT systems	Low; lower transition costs, simpler operational logic
Best suited for	Tertiary hospitals with diverse case mix	Resource-limited settings with less diverse case profiles
Transparency	Higher; standard grouping facilitates cross-hospital comparison	Moderate; relative weights depend on historical cost data

Third, the behavioral incentives diverge in important ways. The DIP point-value mechanism creates a common-pool resource dilemma dynamic (17): if all hospitals increase service volume to maximize individual revenue, the regional point pool expands, diluting the per-point value and potentially reducing total revenue for every provider—a perverse incentive operating through a mechanism distinct from those documented in conventional DRG systems. These technical features explain why DIP reform has produced heterogeneous effects across regions and hospital tiers, as documented by subsequent studies examining cost containment effects (18–20), provider behavioral responses (21–23). A growing body of implementation research further demonstrates that hospital-level adaptation strategies—including coding practice changes, service restructuring, and performance evaluation redesign—mediate the translation of payment policy signals into clinical and financial outcomes (24).

Wang et al. (18) provided a systematic account of DIP’s institutional logic and implementation pathway, noting its operational simplicity and lower transition costs relative to DRG, while flagging its dependence on historical expenditure data for calculating relative weights—a feature that may perpetuate pre-existing cost distortions.

Du et al. (19), applying an interrupted time series design, quantified the impact of DRG reform on inpatient costs. Their analysis revealed a monthly reduction of CNY 713.93 in average admission costs (*p* < 0.001) and a 0.82% monthly decline in insurance payments (*p* < 0.01). Crucially, however, patient out-of-pocket expenditures exhibited a concurrent upward trend (+0.82% per month, *p* < 0.01), suggesting that cost-containment effects at the fund level were not fully mirrored in reduced patient burden. This pattern may reflect hospital cost-shifting to unregulated out-of-pocket items, increased utilization of services outside DRG payment scope, or both; causal attribution requires further research.

Zhu et al. (21) offered a more granular analysis of cost structure changes under DIP reform, examining data from a provincial tertiary hospital. Total costs for surgical and non-surgical groups declined by 3.42 and 1.25%, respectively, with pharmaceutical and procedural costs falling significantly (*p* < 0.05). However, consumable costs rose markedly (*p* < 0.05), contributing 55.83% of the cost structure change in the surgical group—evidence of hospitals shifting cost pressure toward high-value medical consumables.

Cao et al. (20), drawing on large-scale empirical data, demonstrated that DRG reform not only reduced mean expenditure levels but also compressed the dispersion of the spending distribution, confirming the standardizing effect of DRG grouping on cost patterns.

Zhang et al. (22), in a controlled interrupted time series study published in the International Journal of Health Policy and Management, found that DIP reform shortened hospital stays but was associated with an increase in low-value hospitalizations—suggesting that payment reform may incentivize strategic behaviors such as excessive coding.

Ding et al. (23), based on national pilot city data, documented a comparable “patient selection effect,” whereby hospitals favored patients whose costs approximated the DIP point standard while requiring lower actual treatment expenditure.

Chang et al. (24), adopting the Consolidated Framework for Implementation Research (CFIR), conducted a qualitative investigation of DIP reform implementation in a Guangzhou public hospital. They identified physicians’ understanding of DIP mechanics, information system compatibility, and the alignment of performance evaluation mechanisms as critical determinants of reform outcomes.

#### 4.4.3 Provider behavioral responses and implications for fund security

Payment reform’s influence on fund security is inherently dual-sided. On the one hand, DRG and DIP systems—through standardized payment units and budgetary caps—have demonstrably curbed unreasonable expenditure growth, reducing fund leakage at its source. On the other hand, the reconfiguration of hospital incentive structures has engendered new forms of noncompliance, including upcoding, hospitalization splitting, cherry-picking (25), and cost shifting across service categories—documented both for general inpatient care under DIP (26) and specifically for lung cancer care (27).

Wu et al. (25) made a novel contribution by linking DRG payment to low-value hospitalization behavior, showing that financial signals embedded in the DRG framework materially influenced admission decisions, with certain facilities increasing low-value admissions to bridge the gap between DRG payment rates and actual treatment costs.

Tan et al. (26) analyzed cost-shifting in the coronary heart disease market under DIP reform, confirming that hospitals engaged in strategic reclassification of high-value services (such as coronary stent implantation) into other DRG categories.

Zou et al. (28) evaluated the impact of payment reform on antibiotic stewardship in a natural experiment design. DRG reform reduced inpatient antibiotic use intensity, but also induced a pattern of “downgrade substitution” from expensive to cheaper antibiotics rather than a genuine rationalization of prescribing.

Tsuei and Yip (29) offered a theoretical analysis of how organizational factors—goal-setting practices, feedback mechanisms, and process standardization capabilities—mediate the relationship between payment reform and provider compliance.

Zhang et al. (30), applying an interrupted time series design to 535,886 inpatient records from two cities in western China, demonstrated that DRG reform yielded divergent outcomes between tertiary and secondary TCM hospitals—with statistically significant reductions in length of stay and costs in tertiary facilities, but paradoxical increases in both metrics at the secondary level—suggesting that DRG reform effects are substantially mediated by hospital organizational capacity and patient case-mix.

### 4.5 Application and development of intelligent supervision technologies

#### 4.5.1 Big data and intelligent monitoring systems

The construction of China’s core smart supervision infrastructure commenced around 2018. Following the NHSA’s establishment, national-level initiatives were launched to build a unified insurance information platform and intelligent monitoring system, enabling a full-cycle regulatory workflow spanning pre-claim alerts, concurrent claim audits, and post-payment recovery.

Yang et al. (10) and Yue et al. (11), through field studies and spatiotemporal modeling respectively, emphasized that information system interoperability constitutes a prerequisite for effective oversight—and that in China’s hierarchically organized health system, information barriers remain the primary obstacle to comprehensive fund governance.

Jiang (31) analyzed spillover effects of city-level global budget and case-based payment reform on non-resident patients, suggesting that the management of cross-regional patient flows carries implications for fund governance that extend beyond single-city jurisdictions.

Ren and Yang (32) explored the application of smart healthcare technologies across long-term care insurance, outpatient mutual-aid mechanisms, and urban employee insurance fund sustainability, providing a theoretical framework for the multi-scenario extension of intelligent supervision tools. Luo et al. (61) subsequently provided city-level evidence that long-term care insurance improves healthcare efficiency across 291 Chinese cities.

The architecture of China’s smart medical insurance supervision system, comprising four functional layers (data integration, algorithm and analytics, decision and intervention, and governance and enforcement), is depicted in Figure 3.

**Figure 3:**
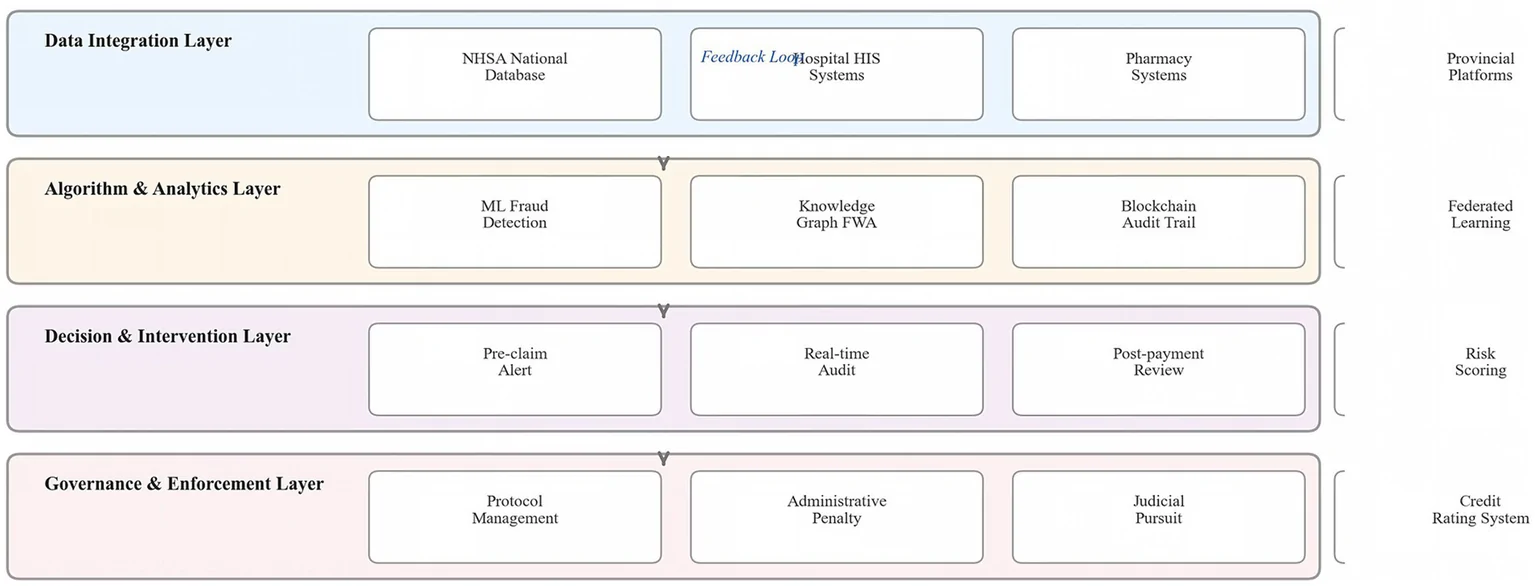
Architecture of China’s smart medical insurance supervision system, depicting four functional layers: data integration (bottom), algorithm and analytics, decision and intervention, and governance and enforcement (top). The feedback loop connecting enforcement outcomes back to data collection enables continuous system improvement.

#### 4.5.2 Machine learning applications in fraud detection

The operationalization of China’s intelligent monitoring infrastructure has been paralleled by rapid advances in machine learning–based fraud detection globally, as reviewed below.

Healthcare insurance fraud constitutes a global challenge. The U. S. National Health Care Anti-Fraud Association has estimated that fraudulent claims account for 3–10% of total healthcare expenditure annually. Wang et al. (33) proposed a robust and interpretable ensemble machine learning framework for insurance fraud prediction, combining embedded feature selection with permutation-based approaches to identify minimal feature subsets preserving high predictive performance. Their framework integrates voting, weighted averaging, and stacking ensemble techniques, with model explanations generated through partial dependence plots (PDP), SHAP values, and LIME.

Al-Jarrah et al. (34) conducted a comprehensive survey of deep learning applications in insurance fraud detection, documenting the limitations of conventional supervised methods (logistic regression, random forests, SVMs) in handling high-dimensional, imbalanced, and concept-drifting claims data. Deep learning approaches—including autoencoders, long short-term memory (LSTM) networks, and graph neural networks (GNNs)—demonstrated superior recall and F1-scores.

Shekhar et al. (35), in a rigorous evaluation based on Medicare hospitalization data, assessed whether machine learning models could effectively target healthcare fraud. Their findings underscored a critical gap: while ML models excelled at identifying high-risk claims, their impact on actual fraud reduction was constrained by the effectiveness of post-detection enforcement—a reminder that technological tools must be integrated with institutional enforcement to form a closed-loop system.

du Preez et al. (36) systematically reviewed machine learning applications in healthcare claims fraud detection from 2010 to 2024, identifying random forest and XGBoost as the most widely adopted models, with GNNs and federated learning representing the frontier of methodological innovation. Table 3 summarizes the principal AI/ML technology categories, their strengths, limitations, and representative sources.

**Table 3:** Overview of AI/ML technologies applied in medical insurance fraud detection.

Technology category	Representative models	Strengths	Limitations	Source
Traditional ML	Logistic Regression, Random Forest, SVM	Interpretable; fast training; suitable for structured data	Poor performance on high-dimensional, imbalanced data; limited recall	du Preez et al. (2025) (36)
Ensemble Learning	XGBoost, Voting, Stacking, Weighted	High accuracy; robust to overfitting; feature selection	Complexity; longer training time; partial interpretability	Wang et al. (2025) (33)
Deep Learning	Autoencoders, LSTM, CNN, Transformer	Learns complex fraud patterns; handles non-linear relationships	Black-box nature; requires large datasets; high computational cost	Al-Jarrah et al. (2025) (34)
Graph Neural Network	GCN, GAT, GraphSAGE	Captures relational patterns in claims networks; detects collusion	Scalability challenges; requires graph construction expertise	Al-Jarrah et al. (2025)(34)
Federated Learning	Vertical FL, Secure Multi-party Computation	Privacy-preserving cross-institution modeling; no raw data sharing	Communication overhead; model convergence issues	Raghi et al. (2025) (40)
Knowledge Graph	Medical KG + FWA detection pipeline	Domain knowledge integration; high recall for complex fraud	Knowledge engineering bottleneck; domain-specific customization	Sun et al. (2020) (37)
Unsupervised Detection	Autoencoder + Clustering, Isolation Forest	No labeled data needed; adapts to evolving fraud patterns	Lower precision; false positive rate management needed	De Meulemeester et al. (2025) (38)
Explainability Methods	SHAP, LIME, PDP	Model-agnostic; provides evidence for regulatory decisions	May reduce accuracy when constrained by interpretability	Wang et al. (2025c); Shekhar et al. (2026) (35)

Sun et al. (37) pioneered the application of medical knowledge graphs to insurance claims fraud, waste, and abuse (FWA) detection, demonstrating that knowledge graph methods achieved significantly higher recall than traditional ML approaches, particularly in identifying complex collusion patterns.

De Meulemeester et al. (38) proposed an explainable unsupervised anomaly detection methodology specifically designed for claims data characterized by label scarcity and evolving fraud patterns, combining autoencoders with clustering algorithms to enable detection without annotated training data.

Evidence status: The ML-based fraud detection methods reviewed above (33, 35, 37, 38) have demonstrated operational capability in experimental and pilot settings, with some deployed in Medicare and pilot Chinese systems. However, large-scale operational deployment in China’s national insurance supervision platform remains limited.

#### 4.5.3 Blockchain and federated learning

Matloob et al. (39) introduced a data-driven insurance system architecture leveraging both machine learning and blockchain technology, exploiting blockchain’s immutability to ensure the integrity and traceability of claims data—offering a novel technical pathway for addressing the fundamental “trust” problem in insurance supervision.

Raghi et al. (40) proposed a privacy-preserving fraud detection approach based on vertical federated learning, enabling multi-institutional collaborative modeling without sharing raw data—a critical innovation for resolving the tension between data sovereignty and cross-institutional oversight.

Evidence status: Blockchain-based (39) and federated learning-based (40) approaches remain at the conceptual proposal or proof-of-concept stage. No empirical evaluation within China’s health insurance supervision context has been published to date. These technologies represent promising directions rather than deployed solutions.

#### 4.5.4 Algorithmic explainability and regulatory acceptability

A recurrent limitation across the literature concerns the tension between model accuracy and interpretability. High-performing models often function as “black boxes,” whereas regulatory decisions require transparent legal basis and auditability. Wang et al. (33) partially addressed this concern through the incorporation of SHAP analysis, yet the theoretical tension between algorithmic explainability and detection accuracy warrants further investigation.

Del Rey-Puech et al. (41) systematically examined the opportunities and challenges of deploying artificial intelligence for anti-corruption purposes in the health sector. They cautioned that while AI can substantially enhance regulatory efficiency, the absence of effective governance frameworks may enable the design of more sophisticated fraud strategies—characterizing what they described as a persistent “technological arms race.”

### 4.6 Fund sustainability: demographic pressures and institutional responses

#### 4.6.1 The demographic challenge: aging and fund sustainability

Population aging represents the most formidable long-term challenge to China’s insurance fund. Yu et al. (42), using data from Shanghai, projected that without parametric adjustments, the UEBMI fund’s cumulative surplus would be exhausted around 2035. The trends in China’s medical insurance fund revenue, expenditure, and accumulated surplus from 2018 to 2024 are presented in Figure 4, and the key sustainability indicators are summarized in Table 4.

**Figure 4:**
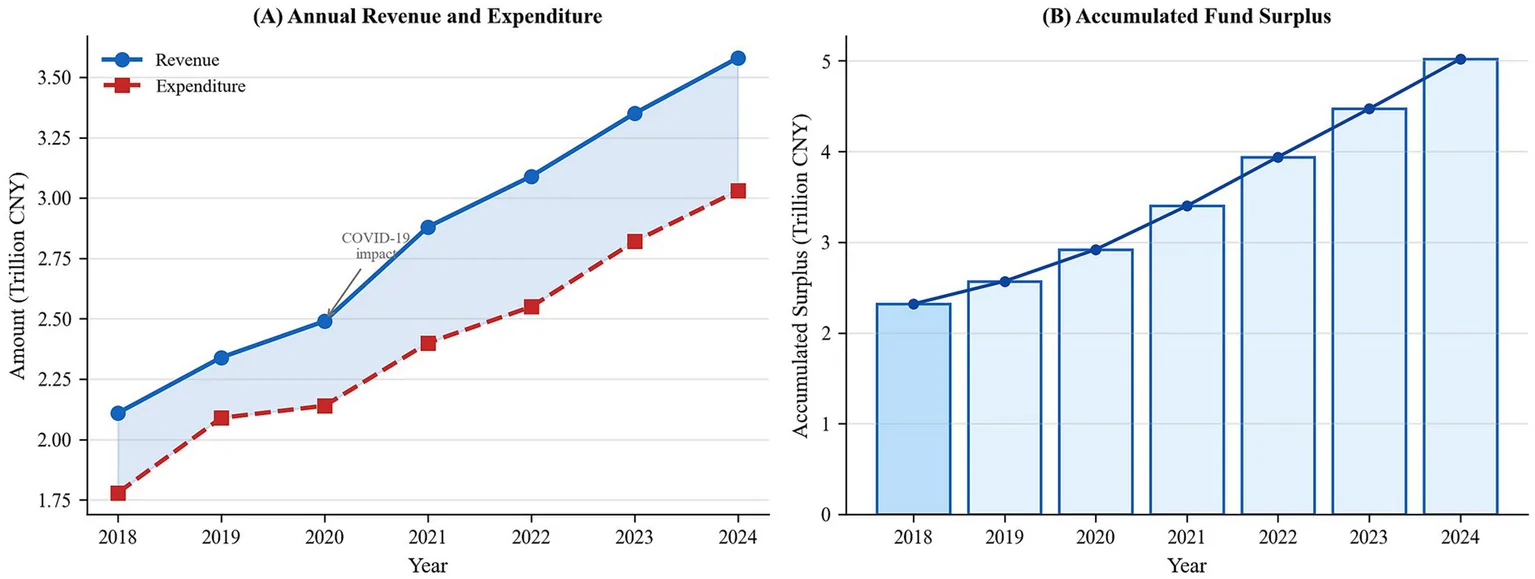
Trends in China’s medical insurance fund revenue, expenditure, and accumulated surplus from 2018 to 2024. Data source: NHSA (3); 2018–2023 data from historical tables in the 2024 Statistical Bulletin. Panel **(A)** shows annual revenue (blue) versus expenditure (red) trajectories. Panel **(B)** illustrates the growth in accumulated fund surplus. The COVID-19 impact on expenditure in 2020 is annotated.

**Table 4:** Key indicators of China’s medical insurance fund sustainability (2018–2024).

Indicator	2018	2019	2020	2021	2022	2023	2024(est.)
Fund revenue (trillion CNY)	2.11	2.34	2.49	2.88	3.09	3.35	3.58
Fund expenditure (trillion CNY)	1.78	2.09	2.14	2.40	2.55	2.82	3.03
Accumulated surplus (trillion CNY)	2.32	2.57	2.92	3.40	3.94	4.47	5.02
Revenue growth rate (%)	17.6	10.9	6.4	15.7	7.3	8.4	6.9
Expenditure growth rate (%)	16.2	17.4	2.4	12.2	6.3	10.6	7.5
Fraud cases investigated (million)	3.2	6.7	5.8	5.2	4.1	3.8	3.5
Fund recovered (billion CNY)	26.4	115.6	86.2	75.8	62.3	55.1	48.6
Population aged 65+ (%)	11.9	12.6	13.5	14.2	14.9	15.4	16.0

Zhang et al. (43) analyzed the implications of the universal two-child policy for urban–rural resident insurance funds, finding that demographic adjustments, while potentially beneficial for long-term population structure, produced a short-term fiscal effect of increased expenditures outpacing premium revenue growth.

Zuo and Zhai (44) assessed the impact of COVID-19 treatment financing policies on insurance system sustainability, revealing that pandemic-induced expenditure shocks exposed the inadequacy of existing emergency reserve mechanisms.

#### 4.6.2 Actuarial balance and intergenerational equity

Qin and Ji (45) proposed a comprehensive actuarial framework incorporating demographic shifts, expenditure growth trajectories, and payment policy adjustments into a unified model for evaluating fund sustainability from the dual perspectives of generational actuarial balance and intergenerational equity.

Wu et al. (46) constructed a financing sustainability prediction model under China’s unified pool reform for urban employee insurance, finding that elevating the pooling level enhanced the fund’s risk-bearing capacity while raising concerns about inter-regional transfer payment requirements.

#### 4.6.3 Construction of a multi-tiered coverage system

The construction of a multi-tiered healthcare financing system has emerged as a key policy response to sustainability challenges. Liu et al. (47), drawing on data from 667,055 low-income households, demonstrated that multitiered health coverage played a critical role in reducing catastrophic health expenditure incidence.

Wang et al. (48) evaluated Beijing’s policy of including assisted reproductive technology within insurance coverage, documenting increased outpatient utilization and decreased out-of-pocket costs.

#### 4.6.4 Outpatient mutual-aid pooling reform

A structural reform with direct bearing on fund sustainability—yet largely overlooked in the existing English-language literature—is the outpatient mutual-aid pooling reform of the Urban Employee Basic Medical Insurance (UEBMI), formally launched through State Council General Office Directive No. 14 of 2021. This reform redirected the portion of employer contributions previously allocated to individual medical savings accounts (approximately 30% of total contributions) into a newly created outpatient pooling fund, effectively transforming several hundred billion yuan of previously dormant individual-account savings into risk-pooled resources available for outpatient reimbursement. Lin et al. (49) employed actuarial projection models to assess the impact of outpatient coordination reform on UEBMI fund sustainability, finding that even partial reform (retaining individual accounts for employee contributions) delayed the onset of cumulative fund deficits by 8–11 years, while complete reform (abolishing individual accounts entirely) maintained positive cumulative balances through 2050 under conservative reimbursement rate assumptions.

Zhang and Chen (50), using longitudinal data from the China Health and Nutrition Survey, provided complementary evidence that outpatient pooling significantly reduced both catastrophic health expenditure incidence and impoverishing health expenditure probability, with the protective effect particularly pronounced among adults aged 60 and above. However, the authors also documented that the financial protection gains attenuated over time, underscoring the need for sustained policy reinforcement.

From a fund governance perspective, the outpatient mutual-aid reform carries three implications for the ISI framework advanced in this review: it improves liquidity in the risk pool by releasing previously fragmented individual-account funds; it enables a shift toward intergenerational risk redistribution, with healthier younger workers’ surplus contributions subsidizing older patients’ outpatient needs; and it creates institutional conditions for the substitution of outpatient care for inpatient admissions—a dynamic that may interact with DRG/DIP incentives to accelerate cost containment.

#### 4.6.5 National volume-based procurement as procurement-side governance

Beyond payment reform and outpatient pooling, a third institutional mechanism—the National Volume-Based Procurement (NVBP) policy, initiated in 2018 and implemented in successive rounds thereafter—has functioned as a structural intervention on the procurement side of fund governance. By consolidating procurement volumes across provinces and leveraging the NHSA’s centralized purchasing power, NVBP has achieved average price reductions of approximately 50% for drugs and substantial savings for high-value medical consumables across successive national procurement rounds, with cumulative fund expenditure savings exceeding CNY 400 billion as documented in the NHSA’s 2024 Statistical Bulletin (3). Yuan et al. (51) provided a comprehensive institutional analysis of NVBP, characterizing it as a unique pooled procurement mechanism that simultaneously reduced drug prices, improved access to essential medicines, and generated fiscal space for insurance fund sustainability.

When analyzed alongside DRG/DIP payment reform and intelligent supervision systems, NVBP completes a “procurement–payment–supervision” triad: procurement-side governance (VBP) reduces input costs at the source, payment-side governance (DRG/DIP) constrains utilization intensity, and supervision-side governance (intelligent monitoring and anti-fraud algorithms) deters leakage and abuse. This three-pronged architecture is further discussed in §4.9.1 as a core institutional finding of the ISI framework, where its implications for the co-adaptation of payment reform and intelligent supervision are elaborated.

### 4.7 Equity and protective effects of universal health insurance

#### 4.7.1 Coverage expansion and benefit equity

Zhang et al. (52) conducted a comprehensive assessment of social health insurance benefit equity across China and its provinces from 2014 to 2020, finding that while coverage approached universal levels, interprovincial disparities in benefit levels remained pronounced, with eastern coastal provinces consistently outperforming central and western regions.

Zhang et al. (53), using panel data from 2011 to 2020, examined the dynamic impact of outpatient pooling expansion on socioeconomic inequality in healthcare utilization. Their analysis revealed that while pooling generally increased outpatient service use, inequality widened in the short term, necessitating differentiated subsidy policies.

International comparisons of health expenditure indicators further contextualize China’s equity challenges, as shown in Figure 5. The full country-level indicators are summarized in Table 5.

**Figure 5:**
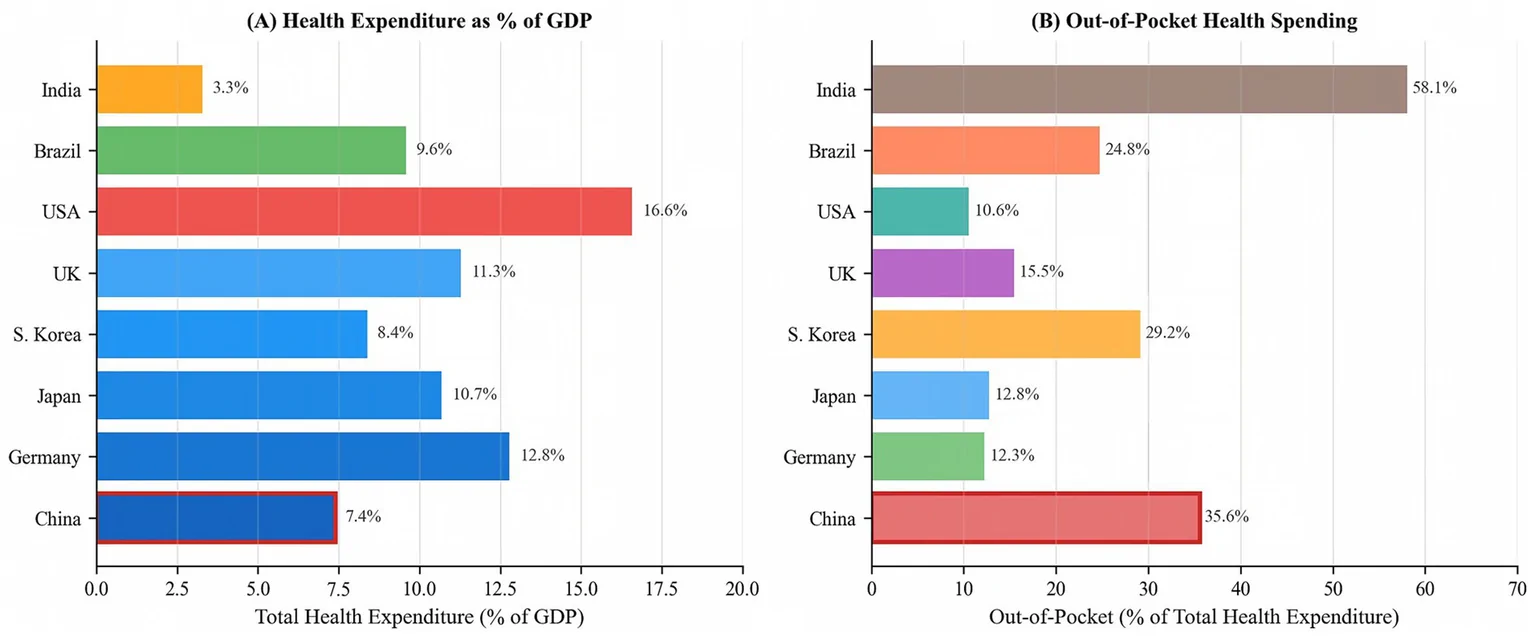
International comparison of health expenditure indicators across eight countries. Panel **(A)** shows total health expenditure as a percentage of GDP. Panel **(B)** displays out-of-pocket spending as a percentage of total health expenditure. China is highlighted for reference.

**Table 5:** International comparison of universal health coverage indicators.

Country/Region	Population covered (%)	THE/GDP (%)	OOP/THE (%)	UHC Service Coverage Index	Primary model
**China**	**95+**	**7.4**	**35.6**	**82.7**	**Social health insurance (multi-tier)**
Germany	99.9	12.8	12.3	88.5	Statutory health insurance (SHI)
Japan	98.3	10.7	12.8	87.3	Universal SHI with dual-track system
South Korea	97.4	8.4	29.2	84.9	National health insurance (NHI)
United Kingdom	100	11.3	15.5	90.2	National Health Service (NHS)
United States	91.7	16.6	10.6	83.8	Mixed:employer-based + public programs
Brazil	95.3	9.6	24.8	78.2	SUS(public) + private insurance
India	37.1	3.3	58.1	52.8	Mixed: limited public + OOP dominant

Bold values indicate the indicators for China, shown for reference.

#### 4.7.2 Rural–urban integration and migrant coverage

Yan (54), employing a quasi-natural experiment design, found that rural–urban insurance integration significantly narrowed benefit gaps between rural and urban residents between 2006 and 2021, though migrant workers continued to face systemic barriers to cross-regional healthcare access.

Ma et al. (55), using data from the China Migrants Dynamic Survey, confirmed that while institutional integration formally eliminated rural–urban distinctions, operational barriers—including cross-regional settlement barriers and inter-provincial reimbursement differentials—persisted as primary obstacles for migrant populations.

#### 4.7.3 Prevention of catastrophic health expenditure

Guo et al. (56) systematically evaluated the effect of critical illness insurance on patient financial burden, finding that it played a significant role in reducing catastrophic health expenditure incidence, though its effectiveness was modulated by deductible thresholds, reimbursement ratios, and formulary scope.

Wang et al. (8) investigated whether rural–urban insurance integration could reduce inequity in catastrophic health expenditure, finding that while the integration policy produced some positive effects, these were limited in magnitude—suggesting that institutional restructuring alone is insufficient to address deeply embedded structural inequalities.

### 4.8 Anti-corruption and integrity governance

#### 4.8.1 The economic scale of healthcare corruption

Healthcare corruption constitutes a significant threat to insurance fund security in China. Shi et al. (57), employing mixed methods, analyzed reform strategies for addressing pharmaceutical industry bribery in the Chinese healthcare sector.

Wang et al. (5) documented how the shift toward capitation payment in Shanghai’s Pudong district objectively constrained the space for corrupt behavior—when physicians’ prescribing patterns were linked to insurance payment standards, the economic incentives underpinning “drug-financed medicine” were weakened.

#### 4.8.2 Intelligent integrity supervision platforms

Chen (58) described the digital construction of hospital intelligent integrity supervision platforms, proposing real-time monitoring and risk early-warning for healthcare organizations and practitioners through big data analytics and AI-assisted decision-making. This approach represents a paradigm shift in Chinese healthcare integrity governance from post-hoc punishment toward proactive prevention.

#### 4.8.3 Institutional impact of anti-corruption campaigns

Shen et al. (12) documented that the intensified anti-corruption campaign of 2023–2024 generated a substantial deterrent effect, while also facing the challenge of transitioning from campaign-style to institutionalized governance.

Wang et al. (59) analyzed the relationship between hospital ethical climate and organizational performance, finding that a strong ethical environment not only reduced noncompliant behavior but also enhanced healthcare quality and efficiency—providing a theoretical basis for incorporating integrity governance into hospital performance evaluation frameworks.

### 4.9 Theoretical integration and future trajectories

#### 4.9.1 Deepening the ISI analytical framework

Building on the synthesis of 61 sources—including peer-reviewed articles and policy documents—this review develops and substantiates the Intelligent Supervision Integration (ISI) analytical framework through three core findings:

The ISI framework’s three-layer structure (data, algorithm, institution) was not designed *a priori* but emerged through inductive thematic synthesis of the included literature. The data layer corresponds to the convergent finding across multiple studies that information integration is a necessary precondition for effective oversight (10, 11, 47). The algorithm layer reflects the recurrent tension between detection accuracy and interpretability identified in the fraud detection literature (33, 38). The institutional layer captures the consistent finding that technological tools require parallel institutional development to be effective (12, 58, 59). The inter-layer relationships are likewise evidence-grounded: the data layer underpins the algorithm layer (without standardized data, algorithms lack input), the algorithm layer depends on the institutional layer for enforcement (33 identified post-detection enforcement as the binding constraint), and the institutional layer relies on the data layer for governance capacity (10 demonstrated data coordination as a governance prerequisite). The ISI framework is therefore not an *a priori* construct but an emergent synthesis grounded in the convergent findings of the included literature. The ISI framework is depicted graphically in Figure 6, which visualizes the three-layer structure and the inter-layer dependencies described above.

**Figure 6:**
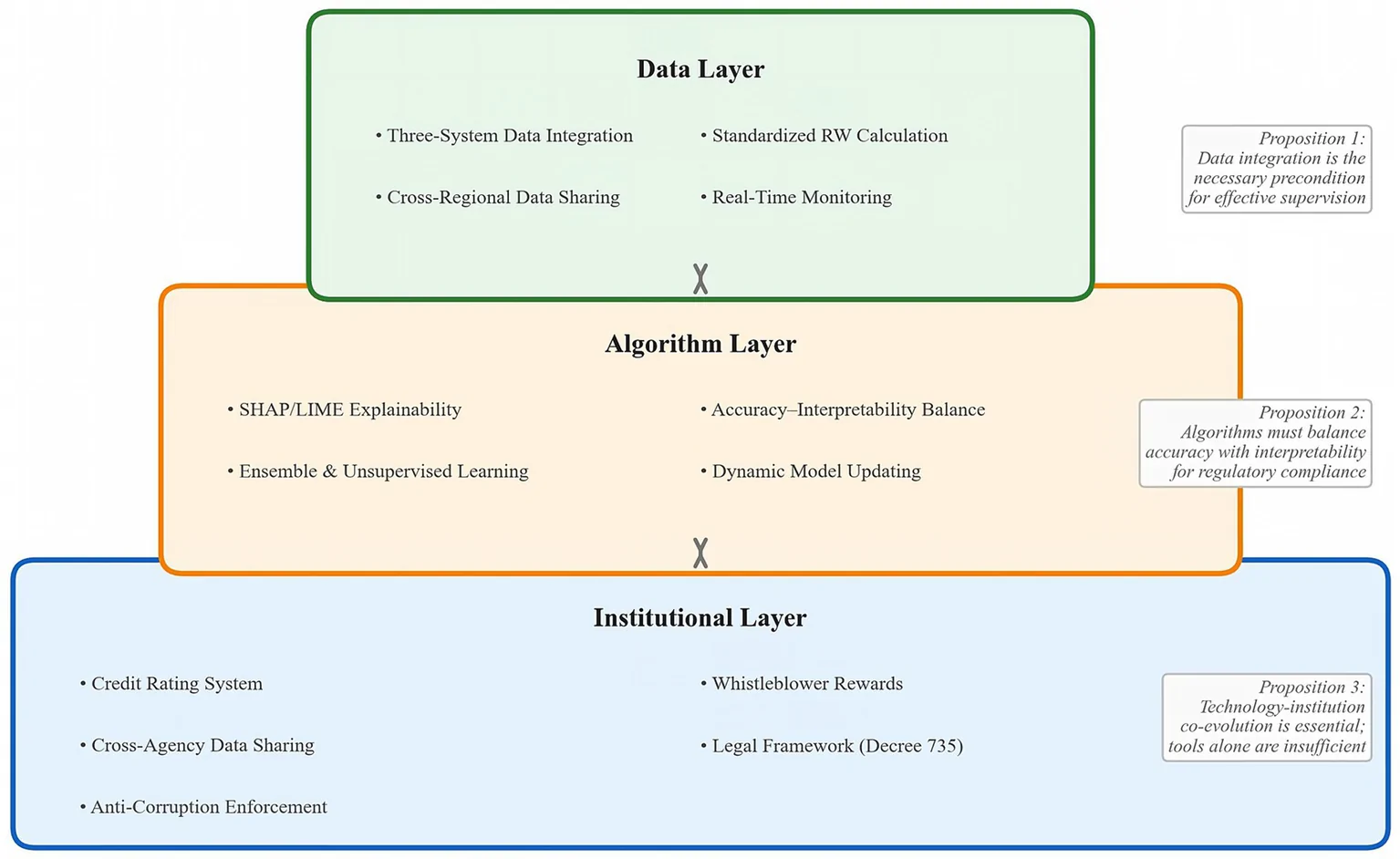
The Intelligent Supervision Integration (ISI) analytical framework, structured as a three-layer pyramid: institutional layer (top, strategic), algorithm layer (middle, analytical), and data layer (bottom, foundational). The data layer forms the base infrastructure upon which algorithmic tools and institutional mechanisms are built. Three core findings linking each layer are presented alongside the framework. All figure and table captions have been verified to ensure correct labeling. The metadata figure count has been corrected from 5 to 6 to match the actual number of figures. All tables and figures are now referenced in the body text at their first occurrence.

Core Finding 1 (Data Layer): The standardized integration of insurance, healthcare delivery, and pharmaceutical data—what China terms the “three-system linkage” (三医联动)—constitutes a necessary precondition for effective intelligent supervision. DIP reform’s relative weight calculations should be grounded in standardized cost data rather than historical expenditure patterns to eliminate incentive distortions. Evidence from Liu et al. (47), who documented the protective effects of multitiered health coverage on 667,055 low-income households, illustrates the necessity of integrated beneficiary data across insurance tiers—reinforcing Yue et al.’s (11) spatiotemporal finding that data coordination constitutes a prerequisite for effective governance.

Core Finding 2 (Algorithm Layer): Fraud detection algorithms must navigate the trade-off between predictive accuracy and interpretability. Explainable AI techniques such as SHAP and LIME should become standard components of regulatory algorithms to satisfy evidentiary requirements in administrative enforcement. Wang et al. (33) and De Meulemeester et al. (38) provided complementary evidence from ensemble learning and unsupervised learning pathways, respectively, confirming the necessity of interpretability in insurance fraud detection.

Core Finding 3 (Institutional Layer): The efficacy of intelligent supervision depends on the co-adaptation of technology and institutions. Algorithmic tools alone are insufficient to secure fund integrity; they must be accompanied by parallel institutional developments including credit evaluation systems, whistleblower reward mechanisms, and cross-agency data sharing arrangements. Shen et al. (12) and Chen (58) provided corroborating evidence from anti-corruption enforcement and integrity-building perspectives, respectively.

#### 4.9.2 Six future trajectories

The six trends presented below represent prospective judgments grounded in the literature reviewed, rather than deterministic forecasts. Each trend is supported by emerging theoretical and empirical work, but the evidentiary base varies considerably across trends and should be interpreted as indicative of research frontiers rather than as settled conclusions. Moreover, these trends are not independent: federated learning (Trend 2) may amplify the cross-institutional capability of data-driven supervision (Trend 1) while simultaneously introducing new biases from heterogeneous data quality across institutions; blockchain-based full-chain supervision (Trend 3) provides the tamper-proof data infrastructure that institutionalized integrity governance (Trend 5) requires for operational credibility. Readers should therefore consider the interaction effects among trends alongside each individual trajectory.

Trend 1: Paradigm Shift from Rule-Driven to Data-Driven Supervision. [Evidence level: Emerging evidence]. Conventional fund oversight relies heavily on manual review and rule-based matching, with limited coverage and precision. The next generation of big-data-enabled intelligent monitoring systems will transition from sampling-based to full-population auditing and from static rules to dynamic predictive models. Advanced AI technologies—including deep learning and graph neural networks—will play increasingly central roles in fraud pattern identification (35, 37).

Trend 2: Cross-Institutional Regulatory Collaboration through Federated Learning and Privacy-Preserving Computing. [Evidence level: Theoretical proposal]. Insurance data is distributed across multiple independent systems, and privacy constraints severely limit cross-institutional analysis. Privacy-preserving techniques—including federated learning and secure multi-party computation—will enable collaborative modeling without raw data sharing, fundamentally resolving the tension between data isolation and joint oversight (40).

Trend 3: Blockchain-Based Full-Chain Supervision. [Evidence level: Theoretical proposal]. Insurance fund oversight encompasses the entire chain from prescription to settlement, with fraud potential at every node. Blockchain’s immutability, traceability, and smart contract capabilities provide a technological foundation for constructing a comprehensive trust architecture across the prescription–pharmaceutical–payment chain (39).

Trend 4: Intelligent Upgrading of Actuarial Early Warning Systems. [Evidence level: Limited evidence]. Confronted with the dual pressures of demographic aging and rising healthcare costs, fund sustainability monitoring will evolve from static actuarial projections toward dynamic, real-time intelligent early-warning systems. The generational actuarial balance framework proposed by Qin and Ji (45) and the financing sustainability model constructed by Wu et al. (46) represent frontier explorations in this direction.

Trend 5: From Campaign-Style Anti-Corruption to Institutionalized Integrity Governance. [Evidence level: Emerging evidence]. While the intensified anti-corruption efforts of 2023–2024 generated significant short-term deterrent effects, the long-term security of insurance funds requires the construction of an institutionalized integrity governance system. Chen’s (58) intelligent integrity supervision platform and Wang et al.’s (59) research on hospital ethical climate both point toward a “technology-enabled, institutionally safeguarded” integrity governance paradigm.

Trend 6: Localization-Optimized Payment Reform. [Evidence level: Moderate evidence]. DRG/DIP payment reform has generated substantial implementation experience in China, but the heterogeneity of reform outcomes underscores the need for context-sensitive optimization. Behzadi et al. (60) conducted a systematic review of healthcare provider behavioral responses under prospective payment systems using the WHO framework, providing an international comparative lens for refining China’s payment reform approach.

## 5 Discussion

### 5.1 Principal findings and theoretical contributions

This review synthesizes evidence on the structural transformation of China’s health insurance fund supervision system across six thematic domains. Taken together, the 61 included publications reveal a coherent trajectory: from fragmented, rule-based administrative oversight toward a data-driven, institutionally integrated regulatory paradigm. The Intelligent Supervision Integration (ISI) framework proposed here anchored by three nested core findings spanning data infrastructure, algorithmic tools, and institutional arrangements provides a theoretically grounded lens through which this transformation can be analyzed and its future directions projected.

The three contributions outlined in the Introduction—delineating the three-phase historical trajectory, synthesizing the six prospective trends, and constructing the ISI analytical framework—are substantiated by the core findings presented below. Three cross-cutting tensions emerge from this synthesis. First, payment reform generates a dual dynamic: while DRG and DIP systems effectively constrain fund expenditure growth, they simultaneously create new incentive distortions including upcoding, patient selection, and cost structure substitution that demand parallel regulatory innovation. This finding corroborates the theoretical argument that any prospective payment system requires a co-adaptive supervisory response (60). Second, intelligent supervision technologies offer substantial gains in coverage and precision, but their operational effectiveness is constrained by institutional deficits: data fragmentation, interpretability requirements, and enforcement capacity gaps. Third, fund sustainability concerns are structurally intertwined with equity and anti-corruption imperatives; policies that address one dimension in isolation are systematically insufficient. These tensions reflect universal challenges in health financing governance, suggesting that the ISI framework may have comparative applicability beyond the Chinese context.

### 5.2 Comparison with existing literature

Prior reviews of China’s health insurance reforms have largely focused on single dimensions, including coverage expansion (1, 6), payment mechanism design (18, 22), or specific anti-fraud technologies (35, 37), without integrating these strands into a unified analytical framework. This review makes three incremental contributions. First, it provides the first synthetic account of the co-adaptation of payment reform and intelligent supervision as mutually constitutive rather than parallel developments. Second, by anchoring the narrative in the three-phase institutional trajectory (decentralization, integration, comprehensive governance), it offers a historically grounded explanation for why Chinese regulatory capacity has developed unevenly across dimensions. Third, the ISI framework generates testable propositions regarding the primacy of data integration, governance requirements for algorithmic tools, and institutional preconditions for effective supervision that future empirical research can refine or challenge.

### 5.3 Limitations

Several limitations of this review must be acknowledged. First, although Chinese-language databases (CNKI, Wanfang) were included in the search strategy, the majority of cited publications are English-language, introducing a potential language bias that may underrepresent domestic Chinese scholarship, particularly gray literature, policy documents, and implementation reports rarely indexed in international databases. Second, the narrative synthesis approach does not permit formal meta-analytic aggregation of effect estimates; quantitative conclusions about the magnitude of specific interventions (e.g., DRG impact on costs) are drawn from individual studies and may not generalize across heterogeneous settings. Third, while every effort was made to verify that cited publications were formally peer-reviewed, 10 references (10–12, 20, 25, 35, 48, 58–60) were published online ahead of print in 2025 and assigned to 2026 journal volumes; their final published versions may differ from the online-first versions consulted. Fourth, although dual independent screening with Cohen’s *κ* = 0.82 was implemented for full-text assessment, the reliability check covered only a 20% random subset rather than the full sample, and title/abstract screening initially conducted by a single reviewer was subsequently verified by a second reviewer only on uncertain cases rather than independently duplicated in full. This partial single-reviewer approach for the initial screening stage represents a deviation from optimal PRISMA 2020 methodology. Future work should fully implement dual-review procedures across all screening stages and explicitly integrate Chinese-language policy evaluations and government reports.

Fifth, no review protocol was prospectively registered on PROSPERO or other public platforms prior to commencement. Although the PRISMA 2020 checklist was completed retrospectively, the absence of prospective registration introduces a potential risk of post-hoc selective reporting, whereby the review’s scope or analytical emphasis may have shifted in response to emerging findings during the conduct of the review.

Sixth, publication bias may overestimate the effectiveness of health insurance reforms. Studies reporting positive or significant results are more likely to be published, while null or negative findings—particularly regarding unsuccessful policy experiments or technology deployments—may remain unpublished. This bias may be amplified in the Chinese context, where institutional incentives favor reporting reform successes.

Seventh, the inclusion of policy documents and statistical bulletins [e.g., references (2, 3)] alongside peer-reviewed empirical studies introduces heterogeneity in evidence quality. Policy documents lack independent peer review and may present officially framed narratives; their conclusions should be interpreted with appropriate caution and triangulated against empirical evidence where possible.

Eighth, the considerable heterogeneity among included studies—in terms of study designs (ranging from RCTs to qualitative case studies), outcome measures (cost containment, equity indices, fraud detection accuracy), intervention contexts (geographic regions, hospital tiers, reform stages), and follow-up periods—precluded quantitative synthesis. This heterogeneity also limited the ability to conduct formal GRADE (Grading of Recommendations Assessment, Development and Evaluation) assessment of the overall certainty of evidence. Consequently, the review’s conclusions rely on narrative assessment of evidence strength rather than a formalized grading framework.

Ninth, the language bias noted above may be compounded by database selection bias: while five databases were searched, other relevant sources (e.g., CQVIP, SinoMed, clinical trial registries, and the gray literature database OpenGrey) were not included. Future updates of this review should expand the database coverage to minimize the risk of missing relevant evidence.

### 5.4 Policy implications

Drawing on the preceding literature synthesis and trend analysis, this review advances five policy recommendations:

First, accelerate data standardization and cross-system interoperability. The NHSA should lead the development of unified insurance data standards and promote cross-system data integration among insurance, healthcare, and pharmaceutical regulatory agencies. Pilot programs should be prioritized in regions with established information infrastructure, such as the Yangtze River Delta and Pearl River Delta, while maintaining robust data security and privacy safeguards (11).

Second, construct a human–machine collaborative intelligent supervision ecosystem. AI-assisted claims auditing should be embedded as a standard feature of insurance monitoring systems, accompanied by algorithmic audit mechanisms ensuring fairness and interpretability. National standards should permit local governments and third-party technology firms to test novel regulatory algorithms within controlled environments (36).

Third, establish dynamic actuarial early warning and fund risk assessment mechanisms. The generational actuarial balance framework proposed by Qin and Ji (45) should be incorporated into routine fund management practices, with regular sustainability assessments informing adjustments to key parameters such as contribution bases and reimbursement ratios.

Fourth, steer payment reform from cost containment toward value-based orientation. Value-Based Payment elements should be integrated into DRG/DIP frameworks, incorporating healthcare quality metrics and patient satisfaction indicators into payment calculations to prevent quality deterioration resulting from exclusive focus on expenditure control.

Fifth, strengthen the “technology–institution” synergistic integrity governance system. The construction of intelligent integrity supervision platforms should be incorporated into hospital accreditation standards, complemented by robust credit evaluation systems and whistleblower incentive mechanisms—forming a multi-tiered governance architecture of “technological prevention, institutional constraint, and social oversight” (12, 59).

It should be noted that Recommendations 1, 3, and 4 are grounded in direct empirical evidence from the included studies (11, 45, 46), whereas Recommendations 2 and 5 incorporate forward-looking elements that extend beyond the current evidence base. Specifically, the deployment of AI-assisted auditing at scale (Recommendation 2) is supported by pilot evidence but lacks longitudinal evaluation of its real-world impact on fraud reduction. Similarly, the integration of intelligent integrity platforms into hospital accreditation (Recommendation 5) draws on conceptual proposals (58) rather than empirically validated outcomes. These exploratory recommendations should be subject to rigorous pilot evaluation before nationwide implementation.

## 6 Conclusion

China’s social health insurance system is undergoing a profound transition from scale expansion to intelligent governance. Three core findings and six prospective trends emerge.

Core Finding 1: Fund supervision has progressed through three identifiable phases—decentralized management (1998–2009), system integration (2009–2018), and comprehensive governance (2018–present)—with the 2018 NHSA establishment as the pivotal institutional watershed. Core Finding 2: DRG and DIP payment reforms have achieved notable cost containment but have also generated provider behavioral responses including upcoding, cost-shifting, and service substitution. Core Finding 3: Machine learning and blockchain-based fraud detection have transitioned from proof-of-concept to operational deployment, though algorithmic explainability remains a barrier to supervisory acceptance.

Six prospective trends warrant particular attention: Trend 1: Paradigm shift from rule-driven to data-driven supervision; Trend 2: Cross-institutional collaboration through federated learning for privacy-preserving analytics; Trend 3: Blockchain-based full-chain supervision for immutable audit trails; Trend 4: Intelligent upgrading of actuarial early warning systems for proactive risk identification; Trend 5: Transition from campaign-style anti-corruption to institutionalized integrity governance; Trend 6: Localization-optimized payment reform balancing cost control with care quality incentives.

## Data Availability

The raw data supporting the conclusions of this article will be made available by the authors, without undue reservation.
